# Immune checkpoint inhibitors for the treatment of MSI-H/MMR-D colorectal cancer and a perspective on resistance mechanisms

**DOI:** 10.1038/s41416-019-0599-y

**Published:** 2019-10-14

**Authors:** Ibrahim Halil Sahin, Mehmet Akce, Olatunji Alese, Walid Shaib, Gregory B. Lesinski, Bassel El-Rayes, Christina Wu

**Affiliations:** Emory University School of Medicine, Winship Cancer Institute, Atlanta, USA

**Keywords:** Immunotherapy, Colon cancer

## Abstract

Metastatic colorectal cancer (CRC) with a mismatch repair-deficiency (MMR-D)/microsatellite instability-high (MSI-H) phenotype carries unique characteristics such as increased tumour mutational burden and tumour-infiltrating lymphocytes. Studies have shown a sustained clinical response to immune checkpoint inhibitors with dramatic clinical improvement in patients with MSI-H/MMR-D CRC. However, the observed response rates range between 30% and 50% suggesting the existence of intrinsic resistance mechanisms. Moreover, disease progression after an initial positive response to immune checkpoint inhibitor treatment points to acquired resistance mechanisms. In this review article, we discuss the clinical trials that established the efficacy of immune checkpoint inhibitors in patients with MSI-H/MMR-D CRC, consider biomarkers of the immune response and elaborate on potential mechanisms related to intrinsic and acquired resistance. We also provide a perspective on possible future therapeutic approaches that might improve clinical outcomes, particularly in patients with actionable resistance mechanisms.

## Background

Colorectal cancer (CRC) is one of the most common cancers in developed countries, with one out of 20 people in the United States of America expected to develop CRC during their lifetime.^1^ It is the second most common cause of cancer-related deaths in men and the third leading cause in women.^2^ Although CRC screening programmes have contributed to decreased incidence and death rates in patients above the age of 50 years in the past decade, the incidence of CRC has been rising in patients younger than 50 years of age^1^ with CRC being the second leading cause of cancer-related deaths in the 40–59 years age group and the third leading cause in the 20–39 years age group.^2^

Mismatch repair (MMR) genes, such as *MLH-1, PMS-2, MSH-2* and *MSH-6*, operate in DNA repair pathways, and the loss of function of these gene products results in MMR deficiency (MMR-D) which is associated with alterations in the size of microsatellites, a phenomenon known as microsatellite instability (MSI). MSI is associated with an increased risk of CRC with unique clinical and pathological features, such as increased tumour mutational burden and higher numbers of tumour-infiltrating lymphocytes (TILs).^3,4^ CRC with MSI can be categorised into two distinct phenotypes: MSI-high (MSI-H) and MSI-low (MSI-L).^5^ MSI-H is historically defined as instability in two or more of the five markers in the Bethesda reference panel (BAT-25, BAT-26, D2S123, D5S346 and D17S250), as detected by PCR, whereas instability in only one marker is considered to be MSI-L.^6^ In more expanded microsatellite panels, instability in more than 30% of the markers is defined as MSI-H and alteration in 10–30% of the markers is considered as MSI-L.^7^ The MSI-H phenotype is frequently associated with downstream frameshift mutations that create a high mutational burden in DNA. Even though MSI-L CRC carries a relatively higher mutational burden than microsatellite-stable (MSS) CRC, the two diseases are phenotypically similar.^5^

MSI-induced frameshift mutations lead to the generation of a significant number of neoantigens, which accounts for the unique phenotypic characteristics of CRC. Unlike point mutations, frameshift mutations lead to alterations in the structure of a whole protein and can create antigenic epitopes that make MSI-H/MMR-D tumours more immunogenic compared with MSS tumours. Accordingly, MSI-H/MMR-D tumours typically display higher numbers of TILs, many of which can be directed against tumour-related neoantigens.^8^

Two inhibitors of the immune checkpoint component programmed cell death-1 protein (PD-1), pembrolizumab (Keytruda) and nivolumab (Opdivo), both of which have been approved by the Food and Drug Administration (FDA) for patients with metastatic CRC with MMR-D or MSI-H, confer a significant survival benefit.^9–11^ Most recently, ipilimumab (Yervoy), a fully humanised monoclonal antibody that blocks cytotoxic T-lymphocyte-associated protein 4 (CTLA-4), has been granted approval by the FDA for use in combination with nivolumab for the treatment of MMR-D or MSI-H CRC patients who were previously treated with chemotherapy.^12^ In the clinical studies of pembrolizumab and the ipilimumab–nivolumab combination, objective responses were 40%^10^ and 54.6%,^12^ respectively, suggesting that there are patients with MSI-H/MMR-D CRC who do not respond to immune checkpoint inhibitors—that is, patients with intrinsic/de novo resistance. Moreover, patients gradually develop resistance to these agents, which suggests that further alterations in the tumour genome and tumour microenvironment (TME) might occur, leading to acquired resistance to immune checkpoint inhibitors.

This review article focuses on the studies that identified the efficacy of immunotherapy in patients with MSI-H/MMR-D CRC, with an emphasis on immune checkpoint inhibitors, and elaborates on the potential mechanisms leading to intrinsic/de novo and acquired resistance to immune checkpoint inhibitors.

## The use of immune checkpoint inhibitors in MSI-H/MMR-D CRC patients

Several studies have investigated the significance of TILs present at the tumour margin in patients with MSI-H/MMR-D CRC.^13^ More favourable survival outcomes and a lower risk of distant metastasis were reported in MSI-H/MMR-D CRC patients with stage III disease whose tumours had higher numbers of TILs compared with MSI-H/MMR-D patients with lower numbers of TILs.^14^ Immunoscore, a classification system based on the extent of CD3^+^ and CD8^+^ T-cell infiltration in the tumour bed, demonstrated the clinical significance of TILs in the recurrence risk of stage I–III MSS CRC patients, suggesting that TILs might have an impact on the prognosis of CRC universally, regardless of MSI status.^15^

These observations led to the exploration of actionable pathways in immune regulation, and clinical trials were designed to assess whether immune checkpoint inhibitors could enhance the anti-cancer of activity of TILs in MSI-H/MMR-D CRC patients.

### Efficacy of immune checkpoint inhibitors

A Phase 2 study investigated the efficacy of pembrolizumab, a humanised IgG4 antibody directed against surface-expressed PD-1, in patients with MSI-H/MMR-D and MSS CRC tumours, and in patients with MSI-H tumours from other sites (non-CRC). Patients enrolled in this study received intravenous (i.v.) pembrolizumab at 10 mg/kg every 2 weeks. The authors reported an objective response rate (ORR) of 40% (4/10) in patients with MSI-H/MMR-D CRC, whereas there was no objective response in patients with MSS CRC (Table 1).^10^ Progression-free survival (PFS) and median overall survival (OS) were not reached in patients with MSI-H/MMR-D CRC at time of analysis with a 12-month median follow up, whereas these values were 2.2 months and 5.0 months, respectively, in patients with MSS CRC. The observed survival benefit was also significant in patients with non-CRC MSI-H/MMR-D tumours. These dramatic results led to FDA approval for the use of pembrolizumab in MSI-H/MMR-D cancers regardless of the histological type of tumour.^16^

**Table 1:** Clinical trials investigating the benefit of immune checkpoint inhibitors in MMR-D/MSI-H CRC patients

Study	Le et al.^10^	Overman et al.^9^	Overman et al.^12^
Design	Phase 2	Phase 2	Phase 2
Number of CRC patients enrolled in the study	A total of 41 patients with various cancer including 10 with metastatic MMR-D/MSI-H CRC	74 metastatic MMR-D/MSI-H CRC patients	119 metastatic MMR-D/MSI-H CRC patients
Agent	Pembrolizumab	Nivolumab	Nivolumab and ipilimumab
Dose	10 mg/kg every 2 weeks	3 mg/kg every 2 weeks	Nivolumab 3 mg/kg in combination with ipilimumab 1 mg/kg every 3 weeks ×4 followed by nivolumab 3 mg/kg every 2 weeks
ORR	40%	31%	55%
PFS	PFS rate at ~5 months (20 weeks) was 78%	PFS rate at 12 months was 50%	PFS rates at 9 months and 12 months were 76% and 71%, respectively
Common adverse effects	Fatigue (32%), rash (24%), diarrhoea (24%), pancreatitis (15%)	Fatigue (23%), diarrhoea (22%), pruritus (10%), rash (10%)	Diarrhoea (22%), fatigue (18%), pruritus (17%), rash (11%), hypothyroidism (14%)
Biomarkers investigated	CD8 and PD-L1 expressions were not predictors of outcome	BRAF, KRAS mutations, PD-L1 expression and Lynch syndrome were not predictors of response	BRAF, KRAS mutations, PD-L1 expression and Lynch syndrome were not a predictor of response

*CRC* Colorectal cancer, *MMR-D* Mismatch repair deficiency, *MSI-H* Microsatellite instability high, *ORR* Overall response rate, *PFS* Progression-free survival, *PD-L1* Programmed death-ligand 1

Nivolumab, a fully humanised IgG4 monoclonal antibody directed against PD-1, has also been investigated in patients with MSI-H/MMR-D CRC. In a Phase 2 trial of 74 patients with metastatic MSI-H/MMR-D CRC, patients received i.v. nivolumab at 3 mg/kg every 2 weeks until disease progression. The authors reported an ORR of 31% (23/74) and disease control rate (DCR) of 69% (51/74), with no treatment-related mortality.^9^ Based on these promising responses, the FDA granted approval of nivolumab for metastatic MSI-H CRC patients in July 2017.

Ipilimumab, a monoclonal antibody targeting CTLA4, was investigated in combination with nivolumab in patients with metastatic MSI-H/MMR-D CRC and the result of this study was reported in 2018. A total of 119 patients received i.v. nivolumab at 3 mg/kg in combination with i.v. ipilimumab at 1 mg/kg every 3 weeks for a total of four doses followed by nivolumab 3 mg/kg every 2 weeks until disease progression.^12^ The ORR and DCR for >12 weeks were 55% and 80%, respectively. In the same study, PFS rates at 9 months and 12 months were 76% and 71%, respectively, suggesting that more effective disease control might be achieved using the combination of ipilimumab and nivolumab compared with nivolumab alone.^9^ The outcomes of this clinical trial led to the accelerated FDA approval of ipilimumab in combination with nivolumab in July 2018 for the treatment of metastatic MSI-H/MMR-D CRC patients.

### Adverse effects of immune checkpoint inhibitors

Although immune checkpoint inhibitors are designed to enhance the immune response against cancerous cells, immune-related adverse events (IRAEs) can occur as a result of an undesired immune response against non-cancerous tissues. Even though immune checkpoint inhibitors can be considered relatively well tolerated, IRAEs may generate significant morbidities and mortalities. Notably, in the study of the combination of nivolumab and ipilimumab, 13% of patients discontinued their therapy due to IRAEs—in particular, autoimmune hepatitis and acute kidney injury.^12^ Diarrhoea, rash, pruritus and endocrinopathies, such as thyroiditis and pancreatitis, were the most common toxicities, both in single-agent anti-PD-1 therapy and combination approaches.^9,10,12^ No treatment-related mortality was reported in these studies. Notably, Overman et al.^12^ reported a 63% ORR in patients who discontinued their therapy due to IRAEs, suggesting that patients who develop IRAEs might still benefit from immunotherapy agents, perhaps even more so than those patients who do not develop IRAEs.^12,17^ Although the exact mechanism remains unclear, IRAEs may be predictor of outcomes in solid tumours including gastrointestinal cancers.^17,18^ IRAEs should be managed in a multidisciplinary manner based on the grade of toxicities and the organ(s) involved.^19^

## Biomarkers for the efficacy of immune checkpoint inhibitors in MSI-H/MMR-D CRC

Immune checkpoint inhibitors are designed to target various regulatory signals on immune and cancer cells, such as PD-1, CTLA4 and programmed death-ligand 1 (PD-L1), so the expression of these target molecules is often used as a reasonable predictive biomarker of a response to treatment in other cancers such as non–small-cell lung cancer,^20^ although this is somewhat controversial due to multiple factors including the methods used to detect PD-L1 expression.^21^ To address these relationships in the setting of CRC, Le et al.^10^ performed a subgroup analysis of MSI-H/MMR-D tumours with high PD-L1 expression (defined as >5%). The authors found, however, that PD-L1 expression in tumours did not predict better survival outcomes in patients with high PD-L1 expression^10^ and a trend to better response was linked to a higher intratumoural CD8^+^ lymphocyte density in baseline tumour samples.

As well as somatic mutations in MMR genes, gene silencing due to hypermethylation of *MLH1*, which is tightly associated with BRAF mutations, might also lead to the development of MSI-H/MMR-D CRC. Investigating the predictive value of BRAF mutations in MSI-H/MMR-D CRC patients receiving nivolumab, Overman et al.,^9^ found that anti-PD-1-based therapy benefited patients similarly in subgroups with or without BRAF mutation. Furthermore, no statistically significant difference in survival was observed based on level of PD-L1 expression (determined low versus high; <1% vs >1%, respectively), the presence of the KRAS mutation or Lynch syndrome, which is the most common cause of hereditary CRC, occurs as a result of germline alterations in MMR genes.^22^ The PD-L1 expression, KRAS and BRAF mutations, and Lynch syndrome status were also analysed in the study of MSI-H/MMR-D metastatic CRC patients treated with the combination of nivolumab and ipilimumab,^12^ but no correlation between clinical response and these factors was identified. However, there was a higher ORR among patients with Lynch syndrome compared with the rest of the cohort (71% versus 48%, respectively).^12^ Notably, approximately 20% of patients who received the combination and 30% of patients who received single-agent anti-PD-1 therapy did not respond to treatment and progressed by 12 weeks of therapy. Although we have not uncovered biomarkers for those patients who do not benefit from immune checkpoint inhibitor therapy in MSI-H/MMR-D cancers, a recent study by Schrock et al.^23^ reported significantly higher tumour mutation burden in responders as compared with non-responders among MSI-H/MMR-D CRC patients who received an immune-checkpoint-inhibitor-based therapy. The authors also categorised their cohort into high and low tumour mutation burden groups and the high tumour mutation burden group had improved PFS as compared with patients with low tumour burden. In that study, the authors examined a relatively small cohort with 22 MSI-H CRC patients using hybrid capture-based next-generation sequencing warranting further studies with larger cohorts to confirm these findings. In another clinical study investigating biomarkers of immune checkpoint inhibitor therapy, more than 300 patients with 22 tumour types including MSI-H/MMR-D CRC tumours were examined by using next-gene sequencing and nanostring platform, and tumour mutation burden and T-cell phenotype were found to be predictors of treatment response.^24^ Notably, the authors in that study used high tumour mutation burden cut-off as 100 mutations per exome as compared with Schrock et al.^23^ and dichotomised their cohort using cut-off point off 37.4 mutations/Mb, indicating the existence of heterogeneity among methodology to identify biomarkers. Other potential biomarkers that remain to be further investigated are the levels of TILs, primary site and metastatic site of tumour, tumour volume and downregulation or overexpression of other immune regulatory signals such as LAG3. Therefore, additional comprehensive studies are warranted to shed more light on biomarkers in this growing area of clinical research.

## Mechanisms of intrinsic resistance to immune checkpoint inhibitors

### MMR gene expression and MSI-H status

MSI-H/MMR-D CRC patients were observed to have fewer metastases to lymph nodes and distant organs compared with patients.^25^ A study found that the presence of TILs with a Crohn’s like lymphoid reaction consisting of discrete lymphoid aggregates was associated with a lower risk of distant metastasis in MSI-H/MMR-D patients,^26^ indicating that the immune response might survey against cancer cells in the local TME, leading to the restraint of early-stage disease. Therefore, it is possible that metastatic MSI-H/MMR-D CRC disease might have already achieved some degree of immunoevasive ability throughout the metastasis process or, alternatively, that these tumours could simply be less immunogenic.^27–29^ It is also important to note that, even though the loss of MMR gene expression is a predictor of the MSI-H phenotype, it might not always be a direct surrogate of MSI-H status, and patients might instead present with MSI-L disease, which is phenotypically similar to MSS tumours.^28^ A study investigating the association of the MSI-H phenotype with Lynch syndrome demonstrated that approximately 36% of patients with Lynch syndrome among all cancer types had an MSS phenotype even though most of these cases were non-CRC (except two patients).^30^ This important finding indicates that MMR gene mutation might not always lead to the loss of MMR gene function, or that MMR gene loss might not always be the driver of carcinogenesis in these patients with Lynch syndrome including CRC. Therefore, next-generation tumour profiling in addition to immunohistochemistry staining could provide important information to further confirm MSI-H status.^31^

### Genetic alterations affecting the immune response

**Fig. 1:**
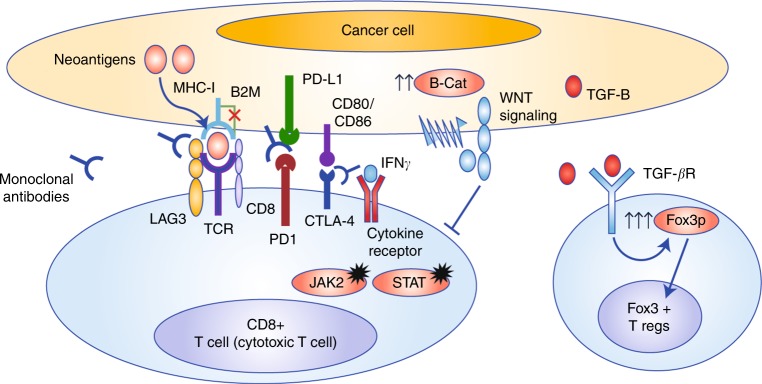
Plausible explanations for immune checkpoint inhibitor resistance in MSI-H colorectal cancers (CRCs). Mutations in β2M and MHC-I result in dysfunction in the antigen presentation process and alterations in JAK2 and STAT lead to impaired interferon signalling. Upregulation of the WNT and TGF-β signalling causes increase in Foxp3^+^ T_REG_ cells and negative regulatory signals on effector immune cells

The exact mechanisms of intrinsic/de novo resistance to immunotherapy in the subgroup of MSI-H/MMR-D CRC patients with metastatic disease are not known but might be explained by biological diversity of the host immune system and tumour biology (Fig. 1).^32^ A study investigated immune evasion mechanisms in CRC patients, including 179 MSI-H/MMR-D CRC patients from the Tumor Cancer Genome Atlas (TCGA), Nurse Health Study and the Health Professionals Follow up Study cohorts, by molecularly characterising tumour samples.^33^ The authors identified multiple genetic alterations leading to immune evasion in patients with MSI-H/MMR-D CRC, mostly in genes related to the immune response, compared with MSS CRCs, in which disruptions in WNT signalling were mostly identified. These genetic alterations in components of the immune response included a biallelic loss of β2 microglobulin (β2M), an MHC class I component, and single-copy loss events in HLA molecules, pointing to antigen presentation machinery defects.^33^ Moreover, the authors also identified alterations in immune-response-related genes that are involved in T-cell responses, B-cell development and natural killer cell function.^33^ Overall, this study identified many potential mechanisms that might primarily be involved in immune evasion and potentially intrinsic/de novo immune checkpoint inhibitor resistance (Table 2).

**Table 2:** Selected preclinical studies investigating de novo resistance mechanisms

Study	Study design	Findings
Grasso et al.^33^	Tumour samples of MSI-H patients (*n* = 179) underwent tumour exome, transcriptome, methylation, and copy-number alteration analyses	Multiple genetic alterations in antigen-presenting machinery including biallelic losses of β2M and HLA genes. GAs were also detected in pathways involving NK cell functions, T-cell response, B-cell development. Upregulated WNT signalling correlated with the absence of T-cell infiltration in the tumour microenvironment.
Riaz et al.^38^	Transcriptome analysis on samples from melanoma patients who received nivolumab therapy	Pre-existing CD8^+^ T-cell clones in the tumour microenvironment predicts response to nivolumab.
Herbst et al.^39^	Immunohistochemical and immunofluorescence evaluation of multiple cancers including two colorectal patients who received atezolizumab	Anti-PD-1 therapy is most effective when the pre-existing immune response is present including T_H_ type1
Li et al.^41^	The computational method was performed to identify the complementarity-determining region 3 sequences of tumour-infiltrating T cells in 9142 RNA-seq samples across 29 cancer types	T-cell diversity correlates with tumour mutation burden and immune response
Michel et al.^47^	Immunohistochemical and immunofluorescence for evaluation of Foxp3 T-cell and CD8^+^ cell infiltration in MSI-H and MSS CRC patients	Increased number of CD8^-^FOXP3^+^ cells found in MSI-H colorectal cancers is paralleled to enhanced CD8-positive lymphocytes to counterbalance the immune response against cancer cells

*MSI-H* Microsatellite instability high, *MSS* Microsatellite stable, *Anti-PD-1* Anti-programmed death 1

### Tumour metabolism and the immune response

A proteogenomic study investigating the metabolism of MSI-H/MMR-D CRC samples identified an inverse association between glycolysis and CD8^+^ T-cell infiltration, suggesting that hypoxic tumours with increased anaerobic glucose catabolism might generate excessive lactate, which is a negative regulator of CD8^+^ T cells.^34^ These data indicate that tumour metabolism, perhaps also tumour volume, might have an important impact on the fate of the immune response in MSI-H/MMR-D CRC patients.^35^

### The influence of the T-cell repertoire on the immune response

The process of clonal evolution, which occurs through T-cell receptor gene rearrangement and clonal selection of cytotoxic T cells in the thymus, serves to eliminate self-reactive clones and influences the diversity of the T-cell repertoire.^36,37^ Mechanistic studies exploring the efficacy of immune checkpoint inhibitors such as those that block PD-1 suggest that these agents are more effective in patients who have pre-existing tumour-reactive effector T cells^38^ and other immune system elements such as T-helper (T_H_) cells.^39^ Therefore, intrinsic/de novo resistance observed in a subgroup of MSI-H/MMR-D CRC patients could be related to a more limited repertoire of cytotoxic T cells^32^ and absence of pre-existing tumour-antigen-reactive T cells.^40,41^ Foxp3^+^ regulatory T (T_REG_) cells have been well studied in immune regulation and their adverse role in cancer progression has been reported in multiple studies.^42^ Current evidence suggests that they suppress the immune response by counteracting cytotoxic T cells via multiple pathways such as secretion of transforming growth factor-β (TGF-β) and its subsequent direct inhibitory action on cytotoxic lymphocytes.^43^ Foxp3^+^ T_REG_ cells are reported to be more abundant in the MSS CRC TME compared with that of MSI-H/MMR-D patients.^44^ The increased relative ratio of intratumoural regulatory T cells to cytotoxic T cells has been identified as an adverse prognostic factor in multiple solid tumours, including in CRC,^45,46^ suggesting that intratumoural T_REG_ cells might be an important determinant in the suppression of the immune response and driving cancer progression. A study investigating the role of Foxp3^+^ T_REG_ cells in MSI-H/MMR-D patients reported their existence in the TME along with CD8^+^ cytotoxic cells,^47^ indicating that there might be a dynamic counterbalance between effector and regulatory T cells which could influence the efficacy of immunotherapy.

### Myeloid-derived suppressor cells

Other populations of the myeloid lineage also have an important role in limiting anti-tumour immune responses. For example, myeloid-derived suppressor cells (MDSCs) limit effective immune responses.^48^ Accumulating evidence suggests that MDSCs impair immune recognition and promote immune evasion in solid tumours via secretion of soluble enzymatic and cytokine mediators, as well as via contact-mediated suppression of T-cell activity.^49^ Further studies might determine whether these negative regulatory cells could be incorporated into biomarker systems such as an immunoscore^15^ to better identify target patient populations who might benefit from checkpoint inhibitors.

### Neoantigens, immunogenicity and mutational load

MSI-H/MMR-D CRC patients are known to have a higher tumour mutational load due to frameshift mutations as a result of MMR deficiency. These major changes in the DNA sequence lead to the formation of neoantigens, which make MSI-H/MMR-D CRC more immunogenic than MSS CRC. This feature brings up an important factor regarding the outcome of mutations in DNA structure: quantity versus quality. Mutations leading to changes in the antigenic structure of proteins are more effective at creating an immune response compared with point-mutation-induced single or limited amino acid changes in the protein, which might fail to induce robust immune responses owing to the preserved antigenic structure and lack of immunogenic epitopes. For example, KRAS point mutations typically show very limited immunogenic activity^50^ and lead to oncogenic activation, which is an important step for the development and progression of multiple solid tumours.^51,52^ Notably, immune checkpoint inhibitors are also known to be more effective against tumours with increased mutational load.^53^ Although mutational load is considered as a surrogate of the presence of various neoantigens, the quantity of mutations might not be directly related to the quality of mutations to generate a robust T-cell response.^54^

## Mechanisms of acquired resistance to immune checkpoint inhibitors

Although the use of immune checkpoint inhibitors is a relatively recent step (with less than 5-years experience) in the treatment of patients with MSI-H/MMR-D CRC, the occurrence of resistance to therapy has been observed.^9,12^ Genomic instability resulting from MSI is a continuous process, leading to the acquisition of new mutations throughout the development and progression of the disease, and some of those mutations might confer acquired resistance to immune checkpoint inhibitors.^55^ Therefore, acquired resistance is likely to have different dynamics and mechanisms to intrinsic/de novo resistance.

### WNT signalling

The WNT/β-catenin pathway is known to mediate multiple pro-growth signals during carcinogenesis and has critical cellular functions including cancer stem cell renewal.^56^ The WNT/β-catenin signalling might also have an important role in immune regulation in the TME.^57^ A study in autochthonous murine melanoma models showed that activation of WNT/β-catenin signalling reduces T-cell infiltration into the TME, thereby reducing the efficacy of immune checkpoint inhibitors.^58^ Another preclinical study with murine melanoma models of 624mel and 888mel melanoma cell lines showed that hyperactivation of the WNT/β-catenin pathway might suppress the effector function of cytotoxic T cells by reducing interferon-γ (IFN-γ) levels (Table 3).^59^ These observations are also consistent with the study discussed above showing that the WNT signalling pathway might impair immune recognition, leading to immune evasion.^33^

**Table 3:** Selected preclinical studies investigating other resistance mechanisms

Study	Study design	Findings
Zaretsky et al.^78^	Paired liquid and tissue biopsies from baseline and after progression in four patients who progressed following initial response to pembrolizumab were molecularly characterised	A truncating β2-microglobulin and loss of function mutations in JAK1/2 with concurrent loss of wild-type allele were identified.
Zhao et al.^76^	Molecular characterisation was performed in tissue samples of a melanoma patient which were obtained throughout multiple recurrences over 6 years	Loss of HLA class I expression in melanoma clones was identified in late recurrent disease leading to T-cell resistance
Spranger et al.^58^	Comparative gene expression profiling was performed in 266 metastatic melanoma patients.	Activated Wnt/β-catenin signalling reduces CD8^+^ T- cell infiltration into tumour microenvironment leading to resistance to anti-PD-1 and Anti-CTLA-4 therapy.
Yaguchi et al.^59^	Mechanistic study in a murine model of melanoma cell lines (B16 and K1735)	Increased Wnt signalling reduces IFN-γ levels leading to progressive immune resistance to which was reversed by a β-catenin inhibitor (PKF115-584)
Chen et al.^64^	Peripheral CD4^+^CD25^−^ naive T cells were treated with TGF-β in vivo and in vitro	TGF-β enhanced *Foxp3* gene expression in TCR-challenged CD4^+^CD25^−^ naive T cells, which led to transition toward regulatory T cells which were carrying highly immunosuppressive potential
Marie et al.^66^	C57BL/6 (B6) and TCRβ/δ-deficient mice were examined for peripheral T regulatory cells quantification	TGF-β1-deficient mice demonstrated peripheral, but not thymic, T regulatory cells. TGF-β1 functions in T regulatory maintenance
Thomas et al.^68^	The effect of TGF-β was investigated in EL4 thymoma cells using a mouse model.	TGF-β suppresses effector function of T cells by actively downregulating the expression of perforin, granzyme A/B and Fas ligand, and thereby leading immune evasion and resistance

*TGF-β* Transforming growth factor- β, *TCRβ* T-cell receptor β chain, *IFN*-γ Interferon- γ, *JAK1/2* Janus kinase 1/2, *anti-PD-1* Programmed cell death protein *1, anti-CTLA4* Cytotoxic T-lymphocyte-associated antigen

Notably, however, there is also evidence that WNT signalling might enhance the generation of self-renewing, multipotent CD8^+^ memory stem cells, which might be important for the creation and maintenance of a potent anti-tumour immune response.^60^ These data suggest that WNT signalling might also confer a beneficial role in effector function and survival of mature T cells.^61^ Therefore, the potential effects of WNT signalling should be further investigated in the context of immune checkpoint responsiveness in MSI-H/MMR-D CRC patients.

### TGF-β

Members of the TGF-β family of cytokines have the ability to mediate complex and diverse cellular functions, including cell cycle control^62^ and angiogenesis.^63^ Besides these direct effects on cancer cells, studies have reported that TGF-β family cytokines might also have important effects on the immune system and on anti-tumour immunity. For example, treatment with TGF-β can induce the conversion of naive CD4^+^ T_H_ cells into T_REG_ cells by upregulating the expression of the *Foxp3* gene (Fig. 1).^64,65^ TGF-β might also be involved in sustaining the immune suppressor function of Foxp3^+^ T_REG_ cells.^66^ In a preclinical study, Ranges et al.^67^ reported a decrease in the generation of cytotoxic T cells in mixed lymphocyte cultures propagated by TGF-β in a dose-dependent manner, leading to deactivation of effector T cells. TGF-β might also suppress the expression of perforin, granzyme A/B and Fas ligand, and thereby neutralise the effector function of cytotoxic T cells.^68^ There is also evidence indicating that reversal of cancer-mediated immune suppression can be achieved by neutralisation of TGF-β.^69^ However, the exact role of TGF-β in MSI-H/MMR-D patients treated with immune checkpoint inhibitors is unclear and needs to be further examined.

### MHC class I molecules

MHC class I molecules have a crucial role in the recognition and presentation of foreign antigens as well as neoantigens created by cancer cells.^70^ A study in MSI-H/MMR-D CRC patients showed that the loss of β2M in tumours from four out of 14 patients leads to impaired MHC class I function.^71^ It is important to note that β2M mutations often occur in the coding microsatellites as a consequence of microsatellite instability making the loss of β2M function almost unique to MSI-H tumours.^72^ These data indicate a multi-step process, which impairs the antigen presentation machinery and leads to the development of resistance^73^ (Fig. 1). At this time, it is unclear if the loss of β2M function in the pre-existing subclones leads to failure of therapy by the selection of clones with antigen presentation machinery defect (immunoselective pressure)^74^ or new clones with antigen presentation machinery defect evolve as a consequence of genomic instability in the setting of MSI throughout the immune checkpoint inhibitor exposure. The data related to the loss of β2M function are also consistent with findings from other solid tumours such as melanoma.^75^ An investigational molecular analysis of a melanoma patient who developed resistance to immunotherapy also showed loss of MHC class I expression in subclones.^76^ The authors also reported an independent β2M mutation leading to dysfunction in the MHC class I complex. Loss of the MHC class I complex and antigen peptide transporters (TAP1/TAP2), which leads to defective antigen presentation, has also been reported in lung cancer.^77^ Finally, a clinical study of four melanoma patients investigating acquired checkpoint resistance identified that the loss of the MHC class I complex due to the mutation of β2M led to an impaired immune response to PD-1-based therapy (pembrolizumab).^78^

### Janus kinases

Janus kinases (JAK) have also been implicated in resistance to immunotherapy. These proteins are members of a family of non-receptor tyrosine kinases that play a growth-promoting role in tumour cells while concurrently regulating immune responses via several mechanisms.^79,80^ Loss-of-function mutations in JAK1/2 are associated with acquired resistance to PD-1 blockade in melanoma patients.^78^ In a mechanistic study, Sucker et al.^81^ reported mutations in JAK2, which led to increased IFN-γ resistance and subsequent anti-PD-1 therapy failure in melanoma patients.

A mutation profiling study of Norwegian and British MSI-H/MMR-D CRC patients suggested that a homozygous loss of JAK1 might be associated with resistance to anti-PD-1 therapy.^82^ However, a retrospective exploratory study in patients with MSI-H/MMR-D CRC treated with nivolumab and ipilimumab identified four patients with a JAK1 loss-of-function mutation that did not appear to impact clinical response.^83^ Taken together, these findings suggest that a biallelic loss of JAK1/2 might be a better biomarker for predicting response to immunotherapy than JAK1/2 mutations. Other proteins in this pathway such as signal transducer and activator of transcription (STAT1/2) function downstream of JAK signalling and are important mediators of IFN-γ signalling.^84^ Mutations in STAT proteins that result in loss of function might also cause impaired IFN-γ signalling and resistance to T-cell-mediated killing in cancer cells.^81,85^

### Epigenetic regulation of T-cell function

Finally, advances in our understanding of the mechanisms of T-cell exhaustion, which abrogates the efficacy of immune checkpoint inhibitors continue to emerge. In particular, there is a high level of appreciation for the role of epigenetics, particularly methylation of genes that influences T-cell phenotype, function and possibly also the durability of response to immune checkpoint blockade. For example, Youngblood et al.^86^ reported that chronic viral infection might lead to demethylation of the PD-1 locus, resulting in T-cell exhaustion and early termination of the immune response. Consistent with this finding, by using whole-genome bisulphite sequencing of antigen-specific murine CD8^+^ T cells, Ghoneim et al.^87^ identified acquired de novo methylation programmes operating during PD-1 blockade that diminish the capacity of T-cell expansion as well as clonal diversity, leading to acquired resistance. Collectively, these data indicate that PD-1 promoter methylation and other epigenetic modifications could constitute key reasons for the failure of immune checkpoint inhibitors, but these potential mechanisms have yet to be investigated systematically in CRC. Advances in single-cell analytic technologies will make this possible and provide data to complement our understanding of the interface between MSI, MSS, other mutations and the epigenetic regulation of T-cell function.

## Conclusions and future perspectives

Immune checkpoint inhibitors are a highly effective therapeutic option for metastatic MSI-H/MMR-D CRC patients with a tolerable toxicity profile. However, intrinsic/de novo and acquired resistance have been commonly observed, and further clinical and translational studies are needed to better understand resistance mechanisms. Moreover, data regarding biomarkers for treatment response and genetic alterations that might revoke the efficacy of immune checkpoint inhibitors are very limited. The use of molecular profiling should be common practice in metastatic CRC regardless of MSI-H/MMR-D status to better characterise underpinnings of disease heterogeneity among MSI-H/MMR-D CRC patients. Notably, immunoscore successfully prognosticated CRC patients with MSS, and this approach could be further investigated for MSI-H/MMR-D CRC patients to assess immune checkpoint responsiveness by better characterisation of immune response using CD3^+^ and CD8^+^ T-cell infiltration in the tumour bed in prospective studies.^15^

Patients with intrinsic/de novo resistance appear to create more challenging clinical situations, as their response to cytotoxic agents might be also relatively limited perhaps due to the lack of significant benefit from fluorouracil-based chemotherapy.^88–90^ In these patients, a thorough characterisation of the TME is essential. For example, the lack of cytotoxic T-cell infiltration, or high ratios of FoxP3^+^ T_REG_ cells:cytotoxic T cells, or the presence of other immune suppressive cells might shed light on resistance mechanisms and enhance therapeutic approaches. In patients with increased FoxP3^+^ T_REG_ infiltration, the use of immune checkpoint inhibitors in combination with agents targeting the T_REG_ population could yield therapeutic effects. Although this approach was not successful in other solid tumours that are hypoimmunogenic, such as pancreatic adenocarcinoma,^91^ a recent study by Fukuoka et al.^92^ identified decreased FoxP3^+^ T_REG_ infiltration in the TME after treatment with a combination of regorafenib and nivolumab and reported an ORR of 29% in MSS CRC patients, warranting further prospective studies with this concept. In patients with no tumour-infiltrating cytotoxic T cells, a thorough the TME analyses for upregulated WNT signalling should be considered. Plausible other approaches such as cancer vaccines or chimaeric antigen receptor (CAR) T cells may also lead to new therapeutic opportunities in tumours lacking cytotoxic T-cell infiltration. Although tumour mutational load has been considered a surrogate for the immune response, the quality of mutations leading to the formation of neoantigens remains a key factor that influences the anti-tumour immune response. Therefore, identification of the diversity of neoantigens and T-cell receptor repertoire might yield a better understanding of primary resistance and provide new therapeutic opportunities for cancer vaccines in patients who have intrinsic/de novo resistance. Molecular profiling of tumour samples from these patients is also essential to discover whether there are mutations in immune response signalling pathways such as IFN-γ. Notably, recent studies revealed that gut microbiome may be linked to immune checkpoint inhibitor response in solid tumour.^93,94^ Even though there has not been consensus in regard to a specific member of the gut microbiome that may enhance the efficacy of immune checkpoint inhibitors, multiple gut bacteria were found to be associated with outcomes when patients treated with anti-PD-1 and anti-CTLA4 immunotherapy. The modulatory effect of the gut microbiome on immune checkpoint inhibitor response may create new therapeutic opportunities and should also be examined in MSI-H/MMR-D CRC patients with intrinsic/de novo and acquired resistance settings.

Patients who acquire resistance after immune checkpoint inhibitor treatment should be evaluated based on their initial therapy. For patients who were treated with only anti-PD-1 therapy, clinical trials investigating a combination of immunotherapies and targeted agents should be considered in the right context (Table 4), such as a combination of checkpoint inhibitors with novel targets. It is important to note that there are many regulatory signals beyond PD-1 that suppress the effector function of cytotoxic T cells, such as LAG3, which might be actionable in patients with anti-PD-1-blockade-resistant disease.^95^ The role of the addition of anti-CTLA4 therapy to anti-PD-1 agents in patients with previous anti-PD-1 exposure is unclear at this time, but there might be a potential benefit in a subgroup of patients, which should be further investigated in prospective studies. Therefore, patients with MSI-H/MMR-D CRC should be strongly considered for clinical trials combining immune checkpoint inhibitors to understand if the addition of another checkpoint inhibitor might be able to overcome acquired resistance. We also recommend obtaining molecular profiling using liquid biopsy or directly from tissue sample if possible, in the appropriate context, to discover genetic alterations that could explain the mechanism of resistance and provide guidance for clinical trial enrollment. For patients harbouring mutations in actionable genes, clinical trials (if available) using a combination of immune checkpoint inhibitors with targeting agents could be strongly considered based on the genetic alteration. For example, patients with defects in homologous recombination DNA repair can be considered for clinical trials combining immune checkpoint inhibitors with poly ADP-ribose polymerase (PARP) inhibitors. In patients who develop resistance mutations in components of the WNT signalling pathway, such as β-catenin, a combined approach using immune checkpoint inhibitors and specific pathway inhibitors should also be considered (Table 4). Therefore, molecular profiling of MSI-H/MMR-D CRC patients might also advance our understanding and trigger future clinical trials combining targeting agents in this patient population.

**Table 4:** Selected ongoing clinical trials investigating immunotherapy in combination with agents targeting resistance mechanisms

Clinicaltrial.gov identifier	Trial design	Rationale/phase of trial/current status	Study group
NCT03095781	Pembrolizumab and XL888 (HSP90 inhibitor) in patients with advance gastrointestinal cancers	Enhancing immune response by upregulating interferon response/Phase 1b/recruiting	Previously treated advanced gastrointestinal cancers
NCT02675946	CGX1321 in subjects with advanced solid tumours and CGX1321 with pembrolizumab in subjects with advanced gastrointestinal tumours (Keynote 596)	Combination of checkpoint inhibitor with Wnt inhibitor to enhance the efficacy/Phase 1b/recruiting	Previously treated advanced gastrointestinal cancers
NCT02947165	Phase I/Ib study of NIS793 in combination with PDR001 in patients with advanced malignancies	Combination of TGF-β inhibitor with anti-PD-1 inhibitor/Phase 1b/recruiting	Advanced solid tumours
NCT03638297	PD-1 antibody combined with COX inhibitor in MSI-H/MMR-D or high tumour mutation burden colorectal cancer	Combining COX-2 inhibitor with an anti-PD1 inhibitor to enhance the efficacy/Phase 2/recruiting	Previously treated MSI-H colorectal cancer
NCT03607890	A study of nivolumab and relatlimab in advanced MMR-D cancers resistant to prior PD-(L)1 inhibitor	Combining anti-LAG3 antibody with an anti-PD1 inhibitor to enhance the efficacy/Phase 2/recruiting	MSI-H colorectal cancer with previous PD-(L)1 exposure
NCT03608046	A study of subcutaneous nivolumab monotherapy with or without recombinant human hyaluronidase PH20 (rHuPH20)	Combining stroma modifying agent with anti-PD1 to enhance the efficacy/Phase 1/recruiting	Previously treated advanced gastrointestinal cancers
NCT03126110	Phase 1/2 study exploring the safety, tolerability, and efficacy of INCAGN01876 combined with immune therapies in advanced or metastatic malignancies	Combining anti-human glucocorticoid-induced tumour necrosis factor receptor with combination of a checkpoint inhibitor to obtain more sustained response	Advanced solid tumours

*HSP* Heat-shock protein*, COX* Cyclooxygenase, *LAG3* Lymphocyte-activation gene 3, *PD-1* Programmed cell death protein 1, *MSI*-*H* Microsatellite instability high, *MMR-D* Mismatch repair-deficient

Finally, it is important to note that the limited number of metastatic CRC patients with an MSI-H/MMR-D phenotype is a challenge for clinical trial design and mandates collaborations between institutions for multicentre clinical studies for this specific patient subset. Therefore, based on potential resistance mechanisms, further prospective collaborative clinical trials are warranted to enroll MSI-H/MMR-D CRC patients who progress on currently approved immune checkpoint inhibitors, which might further improve outcomes for this specific subset of patients.

## Data Availability

Not applicable
